# Probably and Perhaps

**Published:** 1877-04

**Authors:** Thos. K. Beecher


### PROBABLY AND PERHAPS.

THOS. K. BEECHER.

There is much to be said about the blue-glass cure. They alone write absurdly about it who write dogmatically. Nobody knows, for certain, what light is. Nobody understands, perfectly, what life is. Here, then, are brought together two mysteries. He is wise who recognizes that we have very little scientific knowledge as to either.

An event comes to pass—*Whereas, I was sick; now I am well*, says somebody after sitting three weeks in blue sunshine. Now comes the question: What cured that chap? The safest answer is: We do not know and cannot find out, but we are very glad he has got well.

Before we are prepared to affirm or deny anything as to the efficiency of the blue-glass, it is necessary to collate a great number of cases—a larger number than ever *will* be collated. One swallow don't make a summer. The number of people who have bought blue glass and set under it will never be published. But to say that the blue glass did not cure that man who has got well is as absurd as to say that it did.

In a general way we may be quite certain as to the two mysteries, light and life (or health), that they sustain some relation the one to the other. That potato sprout down cellar is sick for want of light. It gets well as soon as it has a daily bath of light. Frequenters of our courts know the look that a man has who has been three months in jail. Light certainly produces some effect upon health. Further, light is of various kinds; it is a compound. When a solar beam is analyzed by the prism, we know that the violet and the actinic or chemical ray, goes toward one end of the spectrum, while the reds and the heat rays, go to the other end. If we sit in the bright daylight to have a picture taken, the face of the sitter reflects nearly all the rays that make white light. But the actinic rays at and beyond the violet of the spectrum do the work of picture-making. The other rays are surplusage.

Whether sunshine affects us by its chemical rays or by its heat rays, is a question which, for one, I cannot answer.

It should surprise no one to find that different portions of the sunbeam affect persons differently. When the equilibrium of health is disturbed, it is not absurd to suppose that by sifting the sunbeam, and using just the portion that is found to be necessary, after careful research, health may be restored. This is conceivable.

But while asserting thus much as being conceivable or even probable, it by no means follows that the blue-glass mania is justified. A sick man might stand at the door of a well-filled drug store and say: There is something in this store that will help me. But this general statement would by no means justify him in going to one particular jar in the store and claiming that its contents would cure him *and everybody else too!*

In the use of drugs we agree that sagacious prescriptions, based on observation and experience, are advisable. In the use, then, of sunlight—as soon as we have gained a knowledge of sunlight, there is little doubt that judicious prescriptions of sun baths may be made.

A word further as to the other mystery—life and health.

There is reason for supposing that all forms of disease will be, sooner or later traced back to a disturbed nervous condition. We know positively that sensation, whether of pain or pleasure, is a matter of nerves. We know that motion, voluntary or involuntary, is a matter of nerves. We know, also, that the ebb and flow of this mysterious tide within us, controls nutrition and the circulation of the blood. We know that emaciation may be brought to pass by cutting nerves. We know that irritation and fever may be brought to pass by stimulation at the nerve centers and the cerebellum.

The theory therefore, is a plausible one, that all disturbance of that equilibrium which we call health is principally a disturbance of those pulses or vibrations or tides which we call nervous.

This being so, it should surprise no one to find that *light* sustains some very positive relations to nervous action; and if to nervous action, then to health.

But all this is *speculation*, plausible speculation indeed, but wholly valueless for practical purposes. If I were a gardener I should certainly experiment on colored glass. Were I a physician I should also experiment. And at what time my own heart and flesh begin to fail me, if I have time and money at command, I shall certainly experiment largely upon the relation of light and heat to the physical comfort of an old man. And when I have found out by experiment that this or that color, or a brindled mix of colors, or the darkness of the violet end, or the warm darkness at the red end of the spectrum, or any part or composition of the whole spectrum, if either one or all these prove a benefit to me, I shall say so. For I end as I began: Life is a mystery, and light is a mystery; he alone is wise who refuses to dogmatize and is content to experiment.

